# Myocardium Assessment by Relaxation along Fictitious Field, Extracellular Volume, Feature Tracking, and Myocardial Strain in Hypertensive Patients with Left Ventricular Hypertrophy

**DOI:** 10.1155/2022/9198691

**Published:** 2022-06-23

**Authors:** Seyed Amir Mirmojarabian, Eveliina Lammentausta, Esa Liukkonen, Lauri Ahvenjärvi, Juhani Junttila, Miika T. Nieminen, Timo Liimatainen

**Affiliations:** ^1^Research Unit of Medical Imaging, Physics, And Technology, University of Oulu, Oulu, Finland; ^2^Department of Diagnostic Radiology, Oulu University Hospital, Oulu, Finland; ^3^Research Unit of Internal Medicine, Medical Research Center Oulu, University of Oulu and Oulu University Hospital, Oulu, Finland

## Abstract

**Background:**

Previous research has shown impaired global longitudinal strain (GLS) and slightly elevated extracellular volume fraction (ECV) in hypertensive patients with left ventricular hypertrophy (HTN LVH). Up to now, only little attention has been paid to interactions between macromolecules and free water in hypertrophied myocardium.

**Purpose:**

To evaluate the feasibility of relaxation along a fictitious field with rank 2 (RAFF2) in HTN LVH patients. *Study Type.* Single institutional case control.

**Subjects:**

9 HTN LVH (age, 69 ± 10 years) and 11 control subjects (age, 54 ± 12 years). *Field Strength/Sequence.* Relaxation time mapping (*T*_1_, *T*_1*ρ*_, and *T*_RAFF2_ with 11.8 *μ*T maximum radio frequency field amplitude) was performed at 1.5 T using a Siemens Aera (Erlangen, Germany) scanner equipped with an 18-channel body array coil. *Assessment.* ECV was calculated using pre- and postcontrast *T*_1_, and global strains parameters were assessed by Segment CMR (Medviso AB Co, Sweden). The parametric maps of *T*_1*ρ*_ and *T*_RAFF2_ were computed using a monoexponential model, while the Bloch-McConnell equations were solved numerically to model effect of the chemical exchange during radio frequency pulses. *Statistical Tests.* Parametric maps were averaged over myocardium for each subject to be used in statistical analysis. Kolmogorov-Smirnov was used as the normality test followed by Student's t-test and Pearson's correlation to determine the difference between the HTN LVH patients and controls along with Hedges' *g* effect size and the association between variables, respectively.

**Results:**

*T*_RAFF2_ decreased statistically (83 ± 2 ms vs 88 ± 6 ms, *P* < 0.031), and global longitudinal strain was impaired (GLS, −14 ± 3 vs − 18 ± 2, *P* < 0.002) in HTN LVH patients compared to the controls, respectively. Also, significant negative correlation was found between *T*_RAFF2_ and GLS (*r* = −0.53, *P* < 0.05). *Data Conclusion.* Our results suggest that *T*_RAFF2_ decrease in HTN LVH patients may be explained by gradual collagen accumulation which can be reflected in GLS changes. Most likely, it increases the water proton interactions and consequently decreases *T*_RAFF2_ before myocardial scarring.

## 1. Introduction

Hypertension is a major public health concern and leading cause of morbidity and mortality globally. Based on international guidelines, hypertension is diagnosed as having systolic blood pressure ≥ 140 mmHg and/or diastolic blood pressure ≥ 90 mmHg [1]. Systemic hypertension is known to increase the risk of developing left ventricular hypertrophy (LVH) and myocardial dysfunction through gene overexpression, protein synthesis, sarcomere rearrangement, and cell metabolism [1, 2]. The progression of LVH is an important determinant of sudden cardiac death and heart failure [2]. Based on left ventricular end-diastolic wall thickness, differential diagnosis between hypertensive LVH (HTN LVH) and hypertrophic cardiomyopathy (HCM) could be challenging [3–5]. Left ventricular global longitudinal strain (LV-GLS) derived from speckle tracking echocardiography (STE) is fast becoming a reliable and reproducible diagnostic tool in the evaluation of hypertrophied myocardium [6, 7]. A limitation of STE, however, is the suboptimal image quality due to insufficient echogenic windows, ultrasound dropouts, and reverberations [8].

Relaxation during radiofrequency pulse forms an endogenous magnetic resonance contrast which is commonly used to study low-frequency interactions between macromolecular content and free water in a wide range of organs and pathologies including musculoskeletal system [9, 10], cancer [11], and myocardial diseases [12, 13]. Many cohort studies have been published on spin-lattice relaxation in the rotating frame (*T*_1*ρ*_) characterizing irreversible myocardium injury. *T*_1*ρ*_ elevation has been found to be significant in the scar tissue at 4-8 weeks postinfarction in swine [14, 15]. Also, comparison of infarct size showed a satisfactory agreement between *T*_1*ρ*_ and late gadolinium enhancement (LGE) [12–16].

The concept of fictitious fields is originated from NMR experiments, where changes in the longitudinal magnetization are generated by a circularly polarized RF field in the xy plane. Based on fictitious field concept, relaxation along a fictitious field with rank *n* (RAFFn) and relaxation time in presence of fictitious field (*T*_RAFFn_) were developed to overcome the specific absorption rate (SAR) problem related to *T*_1*ρ*_ [17, 18]. In RAFFn, the fictitious field is formed by nested sinusoidal amplitude and cosine frequency-modulated RF pulses operating in subadiabatic regime. The presence of fictitious field increases the spin-locking field amplitude leading to reduced SAR, while probing low-molecular motions and generating MRI contrast [19–21]. Different rotating frames can be used to generate the fictitious field and its corresponding relaxation dispersion. The fictitious field rank is determined based on the rotating frame. For example, RAFF is called RAFF2, if the relaxation is occurring in the 2nd rotating frame. *T*_RAFF2_ has been shown to accurately delineate myocardial infarct in a mouse model that is supported by significant correlation between tissue area with elevated *T*_RAFF2_ and Sirius red-stained histology, and *T*_RAFF2_ detected infarct sizes correlated highly with LGE in the mouse model [19]. A recent study of 18 patients with chronic myocardial infarction has shown that *T*_RAFF2_ can be used to delineate focal myocardial fibrosis with clinically acceptable SAR-values [22]. In a previous study, the authors investigated progression of fibrosis in mice with induced hypertrophy using *T*_1*ρ*_, *T*_RAFF2_, and *T*_RAFF3_ and concluded that increases of *T*_1*ρ*_ and *T*_RAFF2_ are most likely from expansion of extracellular volume, with contribution from increased fibrosis content and alterations in exchange rates [20].

The Bloch-McConnell equations are used for modeling the spatial-temporal behavior of the macroscopic magnetization in time-varying external magnetic fields. It is a suitable numerical tool to simulate chemical exchange for various applications including magnetization transfer (MT) or chemical exchange saturation transfer (CEST) imaging [23]. Given a water proton pool and a solute proton pool setup for Bloch-McConnell equations, it is possible to have a realistic simulation of proton chemical exchange in biological macromolecules.

Feature-tracking cardiac MRI (FT-CMR) is a tissue tracking technology for noninvasive assessment of myocardial deformation in research patients [8]. It employs offline tracking of the epi- and endocardial borders upon stacks of 2D cine images. The benefit of FT-CMR lies in quality of cine image and postprocessing operation. This eliminates extra image acquisition and time-consuming protocols which results in easier access and wider availability [24, 25]. There is a considerable amount of literature on prognostic value of FT-derived strain parameters in a wide range of cardiovascular disease such as nonischemic dilated cardiomyopathy, hypertensive heart disease, and hypertrophic cardiomyopathy. Global longitudinal strain (GLS) and global radial strain (GRS) derived from FT-CMR have been demonstrated to be impaired in HCM patients with preserved left ventricular ejection fraction (LVEF) [26]. Recent evidence also shows a significant correlation between global circumferential strain (GCS) and global extracellular volume fraction (ECV), in patients with nonischemic dilated cardiomyopathy [27], in HCM patients with positive and negative late gadolinium enhancement (LGE) [28], and more broadly in a heterogeneous patient population [29]. In addition, previous studies have investigated strain parameters for monitoring the damaged myocardium in HTN LVH patients [30–32].

What is known about hypertrophied myocardium is largely based on left ventricular wall thickness, presence of LGE enhancement, and LV dysfunction detected by impaired strain parameters. Several studies have found a mild increase in native *T*_1_ and ECV in HTN LVH patients without increased LGE [33, 34]. Understanding the water molecular interaction with macromolecules will provide new insights into the progression of LVH in hypertensive patients. In this study, the focus is on myocardial hypertrophy characterization in HTN LVH patients using *T*_RAFF2_ with comparisons to FT-derived strain parameters, native *T*_1_ and ECV. Bloch-McConnell simulation was used to investigate contrast mechanisms.

## 2. Materials and Methods

### 2.1. Study Design and Study Population

The study was approved (159/2018) by the local ethics committee, and each participant provided written informed consent. Prospectively, 9 subjects with HTN LVH (mean age, 69 ± 10 years) and 11 normotensive controls (mean age, 54 ± 12 years) were enrolled between September 2019 and April 2020. All subjects were required to sign informed consent and be in desired physical condition to undergo MR imaging. The definition of HTN LVH was systolic blood pressure ≥ 140 mmHg or diastolic blood pressure ≥ 90 mmHg on at least 2 office readings or taking one or more medications for hypertension followed by maximal LVED − WT ≥ 15 mm in at least one myocardial segment in LGE [33]. Exclusion criteria included significant coronary artery and valvular disease, interventions for septal reduction and pacemaker or defibrillator, other conditions that could cause LV wall thickness, renal impairment with glomerular filtration rate of <45 ml/min/1.73m^2^, and moderate-to-severe systolic function (LVEF < 45%).

### 2.2. MRI Protocol and MRI Reporting

CMR were performed at 1.5 T using a Siemens Aera (Siemens Healthineers GmbH, Erlangen, Germany) scanner equipped with an 18-channel body array coil. Measurements were ECG-gated, and imaging was performed in breath-holds.

RAFF2 waveform was formed using sinusoidal pulse with initial 500 Hz amplitude [17, 18]. RAFF2 pulse trains were created by repeating the refocusing schemes with duration of *T*_*p*_ 2.83 ms 0, 12, and 24 times. To synchronize RAFF2 measurements to the same cardiac phase, a variable delay was added between the *R*-wave and the preparation block. In addition, a crusher gradient was employed to dephase the magnetization in xy plane prior to readout. The RAFF2 imaging parameters were as follows: 6 weighted images (3 with and 3 without an inversion pulse), balanced steady-state free precession (FISP) readout with repetition time (TR) one *R*-*R* interval, echo time (TE) 3.6 ms, matrix size 192 × 156, flip angle 20°, field of view (FOV) 40 × 32.5 cm^2^, and slice thickness 7 mm.

*T*_1*ρ*_ spin-locking pulse consisted of a hard excitation pulse, two halves of spin-lock pulse with reversed phases, and hard refocusing pulse between halves to correct the heterogeneity in the *B*_0_ and transmit *B*_1_ fields [35, 36]. After spin-lock, a hard excitation pulse returned magnetization back to the *B*_0_ direction. Spin-locking times (TSL) were 0, 15, and 30 ms, and the preparation, refocusing, and return pulse amplitudes were 500 Hz. Based on spin-lock durations, a delay was applied to synchronize the readout to a specific time after ECG R-peak. *T*_1*ρ*_ data were fitted using a single monoexponential decay function [35, 36].

For quality control of spin-lock power, RF field amplitude was measured using a 1 ms hard pulse with varying power from 0 to 500 Hz. The measurement parameters were TR one cardiac cycle, TE = 3.6 ms, isotropic in-plane resolution 4.2 mm, flip angle 20°, slice thickness 7 mm, and FISP readout [37].

*T*_1_ mapping was performed using a phase-sensitive variant of the modified Look-Locker inversion recovery (MOLLI) technique with balanced steady-state free precession (b-SSFP) readout. The imaging protocol consisted of a 5 s (3 s)3 s MOLLI sequence scheme with two sets of Look-Locker. This protocol yielded 5 *T*_1_ weighted images over consecutive cardiac cycles during the first inversion recovery. Then, a three-heartbeat gap was followed by 3 *T*_1_ weighted images over consecutive cardiac cycles during the second inversion recovery [38]. The native *T*_1_-mapping parameters were as follows: 8 weighted images, inversion time (TI) from 100 to 5000 ms, TR = 300/TE = 1 ms, isotropic in-plane resolution 1.2 mm, flip angle 35°, and slice thickness 7 mm. The postcontrast acquisition was performed at approximated 10 minutes after the intravenous injection of 0.3 ml/kg gadoteric acid (Grex Medical Oy, Finland) with following imaging parameters: 9 weighted images, TI = 100 − 4000 ms/TR = 300 ms/TE = 1 ms, isotropic in-plane resolution 2.1 mm, flip angle 35°, and slice thickness 7 mm.

LGE images were captured using a 2D single inversion recovery prepared FISP sequence, 10 minutes after the intravenous injection. LGE images were visually evaluated for areas with elevated signal intensity following administration of contrast agent. The imaging parameters were TI = 350 ms/TR = 1100 ms/TE = 1.1 ms, isotropic in-plane resolution 2.1 mm, flip angle 40°, and slice thickness 7 mm.

Multiplanar gated cine CMR was obtained using a standard balanced steady-state free precession readout. Short-axis and long-axis slices with coverage from cardiac base to apex were captured with the following parameters: number of frames 25, number of slices 13-15 depending on the size of heart, TR = 41 ms between single excitations, TE = 1.1 ms, isotropic in-plane resolution 2.1 mm, flip angle 55°, and slice thickness 8 mm.

**Figure 1:**
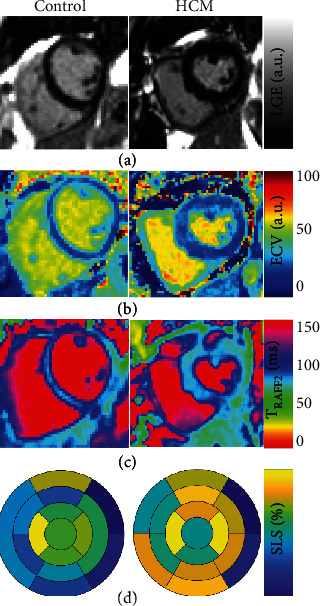
Late gadolinium enhanced image LGE (a), extra cellular volume map ECV (b), RAFF2 relaxation time *T*_RAFF2_ (c), and segmental longitudinal strain (d) showing one case of HTN LVH patients and one from control group using 17-Segment Model (AHA).

**Figure 2:**
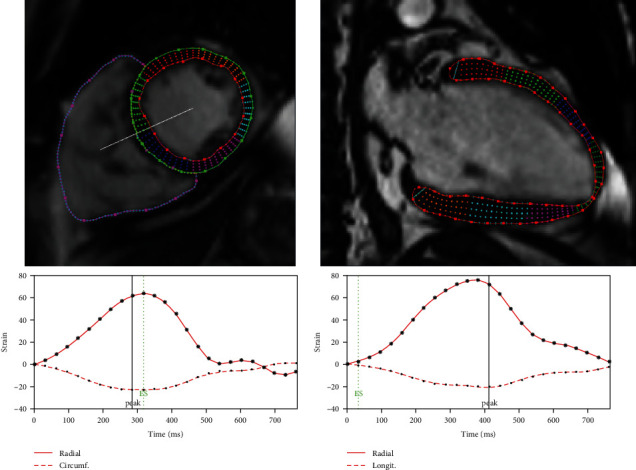
Detected myocardium on cine images using Segment CMR: short-axis and long-axis views. Strain curves are bellow corresponding views.

Global strain analysis was performed using FT-CMR. Cine SSFP images in the short-axis and long-axis views were transferred into commercially available software (Segment CMR, Medviso AB Co, Sweden). Initially, the endocardial and epicardial borders of the myocardial tissue were drawn in each cine MRI image. The software, then, employed a tracking strategy based on nonrigid image registration to compute interframe deformation and consequently to estimate myocardial strain curves [24, 25]. Myocardial global circumferential strain (GCS) and global radial strain (GRS) were calculated from short-axis view and global longitudinal strain (GLS) was derived from long-axis view. To look at strain more locally, we calculated also segmental longitudinal strain shown in Figure 1(d). Peak, upslope rate (US rate) and downslope rate (DS rate) were calculated for strain curves (Figure 2). The myocardial mass and LVEF were also estimated by Segment CMR. LVED-WT was measured from LGE images using Aedes software package (http://aedes.uef.fi/).

### 2.3. Data Analysis

*T*_RAFF2_ and *T*_1*ρ*_ maps were reconstructed using Aedes software (http://aedes.uef.fi/) in MATLAB R2017b platform (Mathworks Inc., Natick, Massachusetts, USA). RAFF2 weighted images were aligned prior to fitting procedure of RAFF2 map. The registration was performed to reduce misalignment artifacts in the final parameter fitting map using Matlab ver. R2017b Registration Estimator toolbox. Standard Siemens protocol was used for *T*_1_ motion correction and relaxation time calculation [38]. Cycle frequency was fitted using single cosine function to obtain *B*_1_ from data with varying preparation pulse duration. Native and postcontrast *T*_1_ maps were aligned using the same registration function as RAFF2. ECV was calculated based on changes within native and postcontrast longitudinal relaxation rates (*R*_1_) and the patient's haematocrit (HCT from 0.37 to 0.47) value with following formula [39]:

(1) $$\begin{array}{c} \text{ECV}=\frac{{\Delta R}_{1\left(\text{tissue}\right)}}{{\Delta R}_{1\left(\text{blood}\right)}}.\left(1-\text{HCT}\right). \end{array}$$

Myocardia were delineated manually, as region of interest (ROI), using Aedes software from *T*_RAFF2_, *T*_1*ρ*_, *T*_1_, and ECV maps and LGE images by one observer. ROIs were traced with margins to stay safely inside the myocardium and avoid partial volume effect. Segment-wise analysis was performed for ECV and *T*_RAFF2_. Imaging data was acquired in mid-ventricular slice, and values therefore reported only respective AHA 17 model segments.

### 2.4. Statistical Analysis

Analysis was performed using SPSS software (version 17.0, SPSS, Inc., Chicago, IL, USA). Continuous data are presented as means ± standard deviation and categorical data as the number (%). The normality was confirmed by the Kolmogorov-Smirnov test. Student's *t*-test was used to compare the mean difference between the HTN LVH patients and controls in all variables. Also, the comparison was conducted in mid-layer segments for ECV and *T*_RAFF2_. Hedges' *g* effect size was calculated as the practical significance representing the standardized difference between means. Association between variables was assessed using Pearson correlation coefficients. An alpha level of 0.05 was used for all statistical tests.

### 2.5. Simulation

The value of *T*_RAFF2_ was estimated using Bloch-McConnell equation in MATLAB (The Mathworks, USA) with a single monoexponential function for the decay of magnetization as described in [17, 18]. A two-pool model was used for proton exchange simulation between free water and hydroxyl group (-OH) representing myocardial collagen type I synthesis. The following parameters were used for OH protons: exchange rate = 1 kHz, concentration = 0.3 − 0.5 M [40], *T*_1_ = 950 ms, *T*_2_ = 50 ms, and chemical shift = 1 ppm.

## 3. Results

**Figure 3:**
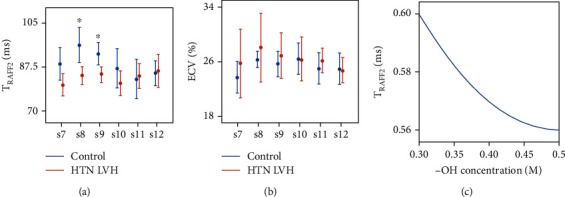
Segment-wise, segments 7–12, (s7-s12), characteristics of HTN LVH patients, and control group given as error bars of (Student's *t*-test) RAFF2 relaxation time (a), ECV (b), and *T*_RAFF2_ Bloch-McConnell simulation (c).

**Figure 4:**
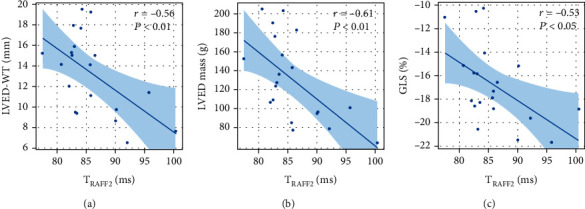
Pearson correlation (*r*), RAFF2 relaxation time (ms) associated with LVED-WT (mm), LVED mass (g), and GLS (%). Generalized linear model fits of the data with 95% confidence bounds.

Bloch-McConnell simulations showed a constant exponential decay in *T*_RAFF2_, from 0.6 to 0.56, when increasing the -OH group concentration from 0.3 M to 0.5 M (Figure 3(c)). Compared to the controls, the HTN LVH patients were older (69 ± 10 vs 54 ± 12 years, *P* < 0.01) with a significantly reduced *T*_RAFF2_ (83 ± 2 ms vs 88 ± 6 ms, *P* < 0.031) and impaired global radial and longitudinal strains (GRS, 41 ± 7 vs 54 ± 6, *P* < 0.001; GLS, −14 ± 3 vs − 18 ± 2, *P* < 0.002). A significant decrease was found in septal wall (segment 8 and 9) of HTN LVH patients using *T*_RAFF2_ (*P* < 0.05) (Figure 3(a)). Substantial difference between the HTN LVH patients and control group can be seen in myocardial strain rates (Table 1); however, GLS and GRS offered the highest practical significances among FT-derived strain parameters with  *g* = 1.53, *g* = 1.92, respectively. *T*_RAFF2_ demonstrated a large practical significance (*g* = 1.02) as observed for strain parameters. In addition, *T*_RAFF2_ was correlated with LVED-WT, LVED mass, GLS (Table 2, Figure 4), and age (*r* = −0.60, *P* < 0.01). The highest correlation was observed between *T*_RAFF2_ and LVED mass (*r* = −0.61, *P* < 0.01).

The HTN LVH patients displayed similar ECV and native *T*_1_ values compared to the control group (ECV, 26% ± 3 vs 25% ± 2; *T*_1_, 975 ms ± 43 vs 948 ms ± 43, respectively). Our analysis demonstrated a significant enlargement in LVED-WT, LVED mass (*P* < 0.01), and borderline LVEF (55 ± 7%, *P* < 0.012) in HTN LVH patients. No noteworthy difference was observed between HTN LVH patients and controls in LGE images (Student's *t*-test  *P* > 0.05). *T*_1*ρ*_ was not correlated with *T*_1_ and heart rate (*P* > 0.05). Altogether, these results show mildly impaired LV systolic function and hypertrophied myocardium with elevated ECV and *T*_1_ in HTN LVH patients (Tables 1 and 2).

## 4. Discussion

In the current study, *T*_RAFF2_ was used to characterize left ventricular hypertrophy in the absence of irreversible myocardial injury in hypertensive patients. The main findings were that *T*_RAFF2_ decreased significantly in HTN LVH patients, *T*_RAFF2_ was statistically associated with myocardium mass, thickness, and LV longitudinal shortening from the base to the apex, and despite significant growth in hypertrophied myocardium mass and thickness, subtle elevation was found in ECV and native *T*_1_ (Table 1, Figure 1).

There are several examples of *T*_1*ρ*_ and *T*_RAFF2_ used for imaging chronic scar tissue in the literature [12–20]. Elevated *T*_RAFF2_ and *T*_1*ρ*_ were reported in fibrotic tissue of TAC-operated mice with mild and severe HCM [20]. Three possible reasons for this elevation were outlined: expansion of extracellular space caused by cell loss and progressive fibrosis, pH shifts, and macromolecular content alteration and proton molecular exchange. In their study, histological analysis was used to confirm fibrotic areas where *T*_RAFF2_ and *T*_1*ρ*_ were elevated. In the current study, *T*_RAFF2_ drew a statistically significant difference between HTN LVH patients and control group. Decreased *T*_RAFF2_ in the hypertensive patient group compared to the control group agrees with the simulation, showing a major decrease in *T*_RAFF2_ most likely by collagen deposition in HTN LVH patients. Furthermore, *T*_RAFF2_ revealed a strong inverse association with myocardium mass, thickness, and GLS (Figure 4). The reason for this rather contradictory result is still not entirely clear, but collagen accumulation without significant increase in water mobility or water content is a reasonable explanation. Relatively low prolonged native *T*_1_ and ECV provided evidence that there were no or only mild increase in extracellular water content. Therefore, it is very likely that increased collagen deposition leads to increased 1H molecular exchange and reduces myocardium *T*_RAFF2_.

Hemodynamic burden is a significant contributory factor to the development of LVH. The increase in LV mass and wall thickness occurs to compensate chronic pressure overload in hypertensive patients. However, it is the cellular and molecular response to sarcomere-gene mutations that induce collagen deposition and fibrotic remodeling [41]. Increased myocardial collagen synthesis leads to expansion of the extracellular space, and consequently, to development of left ventricular hypertrophy. Several studies have shown increased native *T*_1_ and ECV in HTN LVH patients [33, 34]. Our findings are in line with previous results showing a relative increase in ECV (26 ± 3%vs 25 ± 2%) and native *T*_1_ (975 ms ± 43 vs 948 ms ± 43) of HTN LVH patients compared to controls. This result may be explained by the fact that fibrotic replacement is gradual as disease develops from collagen deposition to increased extracellular space.

Our results showed no correlation between *T*_1*ρ*_ and aging which confirms those of Wang et al. who also found that there is no association between *T*_1*ρ*_ and aging in myocardium [42]; however, we observed a significant decrease in *T*_RAFF2_ with increasing subject's age. Aging is a slow but life-long process associated with physiological changes in all organ systems. It increases arterial wall thickening and stiffness due to deposition of collagen and microscopic changes in cardiovascular systems [43]. Therefore, *T*_RAFF2_ changes may partly be explained by the increased water proton interactions due to aging. The Bloch-MocConnell equations were used to simulate chemical exchange as the source of contrast mechanism in *T*_RAFF2_. Our results showed that there is a major difference between simulations (~600 ms) and measurements in vivo (~80 ms). The primary cause of the discrepancy is in numerous relaxation channels in vivo contributing to *T*_RAFF2_, while only proton exchange is resembled with Bloch-McConnell equations.

Several studies have shown impaired GLS in HTN LVH patients without LGE enhancement despite preserved LVEF [30–32]. GLS tracks myocardium deformation in long axis and disproportional LV thickening, while LVEF is a clinical measure of LV function. Neisius et al. reported significant correlation of GLS with LV mass index (*r* = 0.42, *P* < 0.002) and LVEF (*r* = −0.49, *P* < 0.002) in hypertensive patients [32]. In accordance with the research, our result confirms GLS impairment and its correlations with LV mass and LVEF. In addition, our findings showed higher practical significance for GLS and GRS compared to strain rates. This may mean that the limit of myocardial contraction at end-systole is more sensitive biomarker in differentiating HTN LVH compared to the rate of contraction at which deformations occur.

### 4.1. Limitations

The present study has some limitations. A small sample size and single center measurements were the most evident constraints. Also, the study was exploratory in nature with multiple measures being compared and correlated without correction for multiple comparisons leading to the increased risk of false positive findings. Prospective and diverse multicenter studies with the heterogeneous group of patient population are necessary to generalize our findings. Prognostic data are also needed to evaluate the prognostic value of the RAFF and FT-CMR strain in HTN LVH patients. Software packages are another limitation in our work. Since there is no standard protocol used in software packages, different registration framework or FT-CMR software package is possibly to introduce variability into our results. This might affect strain values or out-of-plane movement correction.

## 5. Conclusion

Our results demonstrate a good agreement between decreased *T*_RAFF2_ and myocardial strain in the absence of scar tissue and mild diffuse fibrosis in hypertrophied myocardia. Most likely, augmented collagen content increases the water proton interactions which leads to a decrease in *T*_RAFF2_ of hypertrophied tissue when extracellular space is still close to intact. Both theoretical simulation and empirical evidence support this hypothesis. *T*_RAFF2_ can shed new light on early changes in myocardial pathology with preserved LVEF.

## Figures and Tables

**Table 1:** Characteristics of HTN LVH patients and control group given as mean ± SD and global values (Student's *t*-test).

	HTN LVH (*n* = 9)	Control (*n* = 11)	*P* value	Hedges' *g*
*T*_1_ (ms)	975 ± 43	948 ± 43	ns	—
ECV (%)	26 ± 3	25 ± 2	ns	—
*T*_1*ρ*_ (ms)	60 ± 4	62 ± 7	ns	—
*T*_RAFF2_ (ms)	83 ± 2	88 ± 6	0.031	1.02
GCS	−17 ± 2	−19 ± 3	ns	—
GCS US rate	77 ± 19	93 ± 26	ns	—
GCS DS rate	−94 ± 14	−103 ± 16	ns	—
GRS	41 ± 7	54 ± 6	0.001	1.92
GRS US rate	217 ± 52	249 ± 47	ns	—
GRS DS rate	−209 ± 58	−305 ± 99	0.021	1.10
GLS	−14 ± 3	−18 ± 2	0.002	1.53
GLS US rate	56 ± 9	74 ± 18	0.016	1.17
GLS DS rate	−69 ± 15	−85 ± 11	0.013	1.18
LVEF (%)	55 ± 7	64 ± 6	0.012	1.33
LVED Vol (ml)	141 ± 47	129 ± 26	ns	—
LVED mass (g)	162 ± 29	112 ± 36	0.004	1.44
LVED-WT (mm)	17 ± 2	10 ± 2	0.001	3.35

**Table 2:** Pearson correlation coefficients (*r*) between *T*_RAFF2_, ECV, and strain parameters and global values (^∗^*P* < 0.05, ^∗∗^*P* < 0.01).

	*T*_RAFF2_ (ms)	GCS	GRS	GLS
*T*_1_ (ms)	-0.19	-0.20	0.09	0.32
ECV	0.19	0.17	-0.26	0.06
*T*_1*ρ*_ (ms)	0.38	-0.05	0.29	-0.10
*T*_RAFF2_ (ms)	1	-0.23	0.47^∗^	-0.53^∗^
GCS	-0.23	1	-0.62^∗∗^	0.58^∗∗^
GCS US rate	0.23	-0.60^∗∗^	0.63^∗∗^	-0.43
GCS DS rate	-0.33	0.57^∗∗^	-0.56^∗∗^	0.21
GRS	0.47^∗^	-0.62^∗∗^	1	-0.56^∗^
GRS US rate	0.15	-0.18	0.65^∗∗^	-0.01
GRS DS rate	-0.46^∗^	0.41	-0.76^∗∗^	0.52^∗^
GLS	-0.53^∗^	0.58^∗∗^	-0.56^∗^	1
GLS US rate	0.33	-0.38	0.44	-0.62^∗∗^
GLS DS rate	-0.44	0.58^∗∗^	-0.62^∗∗^	0.78^∗∗^
LVEF (%)	0.21	-0.76^∗∗^	0.73^∗∗^	-0.49^∗^
LVED Vol (ml)	-0.08	0.26	-0.55^∗^	0.031
LVED mass (g)	-0.61^∗∗^	0.16	-0.67^∗∗^	0.61^∗∗^
LVED-WT (mm)	-0.56^∗∗^	0.22	-0.56^∗^	0.67^∗∗^

## Data Availability

The MRI maps and extracted strains parameters used to support the findings of this study were approved by the local ethics committee and so cannot be made freely available. Requests for access to these data should be made to the corresponding author, Timo Liimatainen, timo.liimatainen@oulu.fi.
